# [18F]FDG PET/CT and PET/MR in Patients with Adrenal Lymphoma: A Systematic Review of Literature and a Collection of Cases

**DOI:** 10.3390/curroncol29100623

**Published:** 2022-10-18

**Authors:** Laura Evangelista, Filippo Crimì, Andrea Visentin, Giacomo Voltan, Livio Trentin, Carmelo Lacognata, Diego Cecchin, Filippo Ceccato

**Affiliations:** 1 Nuclear Medicine Unit, Department of Medicine DIMED, University-Hospital of Padova, 35128 Padova, Italy; 2 Institute of Radiology, Department of Medicine DIMED, University-Hospital of Padova, 35128 Padova, Italy; 3 Hematology and Clinical Immunology Unit, Department of Medicine DIMED, University-Hospital of Padova, 35128 Padova, Italy; 4 Endocrinology Unit, Department of Medicine DIMED, University-Hospital of Padova, 35128 Padova, Italy; 5 Radiology Unit, University-Hospital of Padova, 35128 Padova, Italy

**Keywords:** primary adrenal lymphoma, FDG, PET, response to therapy, SUV

## Abstract

Aim. The present study aimed to assess the existing data about Primary Adrenal Lymphoma (PAL) evaluated with FDG PET and to describe a small monocentric series of cases. A systematic analysis (from 2010 to 2022) was made by using PubMed and Web of Science databases reporting data about the role of FDG PET/CT in patients with suspicious or known adrenal lymphoma. The quality of the papers was assessed by using QUADAS-2 criteria. Moreover, from a single institutional collection between 2010 and 2021, data from patients affected by adrenal lymphoma and undergoing contrast-enhanced compute tomography (ceCT)/magnetic resonance (MR) and FDG PET/CT or PET/MR were retrieved and singularly described. Seventy-eight papers were available from PubMed and 25 from Web of Science. Forty-seven (Nr. 47) Patients were studied, most of them in the initial staging of disease (*n* = 42; 90%). Only in one paper, the scan was made before and after therapy. The selected clinical cases were relative to the initial staging of disease, the restaging, and the evaluation of response to therapy. PET/CT and PET/MR always showed a high FDG uptake in the primary adrenal lesions and in metastatic sites. Moreover, PET metrics, such as maximum standardized uptake value (SUVmax) and metabolic tumor volume (MTV), were elevated in all primary adrenal lesions. In conclusions, FDG PET either coupled with CT or MRI can be useful in staging, restaging, and for the evaluation of treatment response in patients affected by PAL

## 1. Introduction

Primary Adrenal Lymphoma (PAL) is an extremely rare form of lymphoma, which accounts for less than 1% of non-Hodgkin Lymphoma (NHL) cases. Although a shared and widely accepted definition is still missing, the term PAL is often relative to patients with the involvement of one or both of the adrenal glands and no prior history of lymphoma, nor concomitant dominant lesions in other organs [1,2]. PAL usually occurs in males within the sixth–seventh decade of life [3] and its clinical presentation is characterized by systemic B-symptoms (fever, weight loss, night sweats), abdominal or back pain and fatigue, respectively in 68%, 42% and 36% of cases. Another common clinical manifestation is adrenal insufficiency (AI), especially in bilateral form, which has been reported in 61% of patients [2]. Diagnostic work-up of PAL includes radiological and nuclear medicine examinations such as contrast-enhanced computed tomography (ceCT), magnetic resonance imaging (MRI) and positron emission tomography PET/CT with 18F-Fluorodeoxyglucose (FDG), although adrenal biopsy is mandatory for histological confirmation and classification. However, biopsy should be performed after excluding catecholamine hypersecretion [4,5]. Prognosis is usually considered poor, due to the lack of reliable serum markers, the aggressiveness of the disease and the rapid progression of PAL, with a high risk of spread to the central nervous system [6,7]. The rarity of this disease, furthermore, could hamper an early diagnosis with several consequences, such as a delay in starting therapies and the lack of identification of potential life-threatening conditions like AI [2].

Considering all these aspects, to achieve a prompt diagnosis, a multidisciplinary approach involving endocrinologists, radiologists, nuclear medicine physicians, pathologists and hematologists is strongly recommended. In this mini-review and pictorial essay, we present five cases of PAL inclusive of nuclear medicine examinations, in accordance with a review of the existing literature.

## 2. Materials and Methods

A literature search (including the period from 2010 to 2022) was performed for available articles that reported data about the role of FDG PET/CT in patients with suspicious or known adrenal lymphoma. Moreover, from a single institutional collection between 2010 and 2021, data from patients affected by adrenal lymphoma and undergoing ceCT/MR and FDG PET/CT or PET/MR were retrieved and described.

### 2.1. FDG PET Acquisition and Interpretation

All patients were required to fast at least for 6 hours and maintain adequate hydration before the scan. Blood glucose levels were measured in all patients before FDG administration. In the case of a fasting glucose level above 200 mg/dl, the FDG PET scan was postponed until a proper therapy was established. FDG injection (3 MBq/Kg of body weight) was performed 60 min before image acquisitions. Two dedicated PET/CT systems (Siemens Biograph 16S or Philips Ingenuity scan) were used. PET/MRI were acquired using hybrid equipment (Biograph mMR^®^; Siemens Healthcare, Erlangen, Germany) with 3T MRI. Data acquired with the CT and MR scan were used for attenuation correction and fused with PET images. PET/CT or PET/MR data were reconstructed using a dedicated commercial workstation along axial, coronal and sagittal views. A physician in nuclear medicine with at least 10-years of experience visually evaluated PET/CT and PET/MR images. Sites of disease, defined as the presence of an abnormal FDG uptake, were annotated in a dedicated spreadsheet. For the post-therapy scan, Lugano Classification was used, based on the Deauville Score [8].

### 2.2. Literature Search

A systematic analysis was made by using PubMed and Web of Science databases. The following keywords were used: “FDG PET/CT” AND “Adrenal Lymphoma”, “PET/CT” AND “Adrenal Lymphoma”, “PET/CT” AND “Lymphoma in the adrenal glands”. Filters such as languages (only English) and humans were applied. The quality of the papers was assessed by using QUADAS-2 criteria (http://www.bristol.ac.uk/population-health-sciences/projects/quadas/quadas-2/, accessed on 27 August 2022). Two independent authors (L.E. and F.C.) analyzed all papers. In the case of discordance, an agreement was reached.

## 3. Results

**Figure 1:**
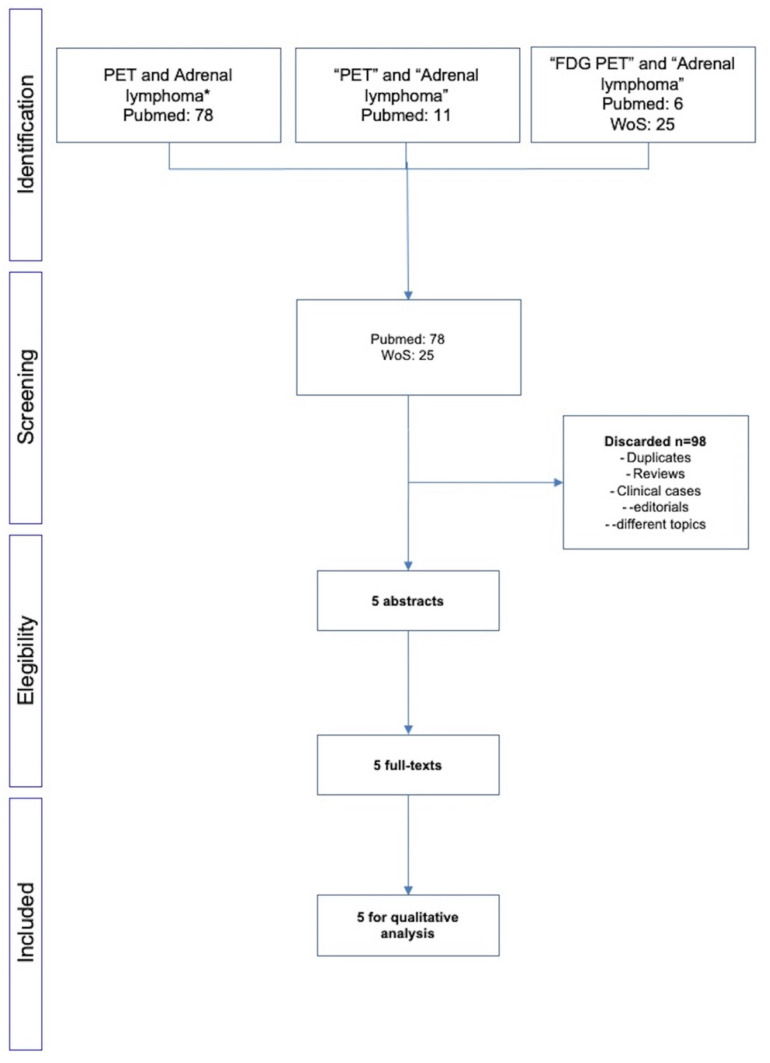
Flow-chart of the literature search. * both Hodgkin and non-Hodgkin lymphoma.

From 2010 to 2022, 78 papers were available from PubMed and 25 from Web of Science. After excluding reviews, letters to the editors, editorials and clinical cases, only five papers meet the inclusion criteria and therefore were considered for the final analysis (Figure 1) [9,10,11,12,13]. The characteristics of the studies are reported in Table 1.

### 3.1. Analysis of Available Papers

Currently, few papers are available about the use of [18F]FDG PET/CT in adrenal lymphoma. Based on the retrieved data, 47 patients were studied, most of them in the initial staging of disease (*n* = 42; 90%). Only in one paper, the scan was made before and after therapy. In many cases, visual and semiquantitative data were used for the interpretation of the molecular imaging [9,11]. In a single paper, a radiomic analysis was employed for supporting the analysis of the images. The quality of papers was reported in Table 2. As illustrated, patient selection and the standard of reference were unclear in some studies [9,10,11]. In the study by Kasaliwal et al. [10], few data were reported about the utility of FDG PET/CT in this setting of disease. The authors stated that FDG PET/CT can be helpful to identify the involvement of regional lymph nodes, other than the adrenal glands and can help to determine the response to therapy and to indicate with high probability the diagnosis of adrenal lymphoma. Laurent et al. [12] used FDG-PET in 10 patients with a suspicion for adrenal lymphoma. The authors found that this imaging modality allowed a more precise evaluation of the PAL extension, by eliminating doubtful sites seen on the CT-scan. This latter concept was also underlined by Altinmakas et al. [11] and Majidi et al. [9]. Moreover, Altinmakas et al. [11] reported that the primary adrenal lymphoma had a high FDG uptake, with the SUVmax ranging between 10.3 and 49.2 (median: 18.6). Similarly, the only patient affected by a T-cell lymphoma had a SUVmax equal to 11.4. Finally, Wang et al. [13] explored the role of radiomics in patients affected by renal (*n* = 11 pts) and adrenal lymphoma (*n* = 8 pts), demonstrating that pretreatment PET-image-based parameters, including uptake indices (SUVmax, SUVmean, MTV, TLG) and texture parameters, were correlated with the survival outcomes. However, no separate analysis for adrenal and renal lymphoma was available.

### 3.2. Clinical Cases

The selected clinical cases were relative to the initial staging of disease (case #1), the restaging (case #2) and the evaluation of response to therapy (cases #3, #4 and #5). The clinical assessment and [18F]FDG PET metric were reported in Table 3 and Table 4, respectively. Moreover, additional information about histological characteristics were reported in Supplementary Table S1. 

#### 3.2.1. Initial Staging of Disease: Case #1

**Figure 2:**
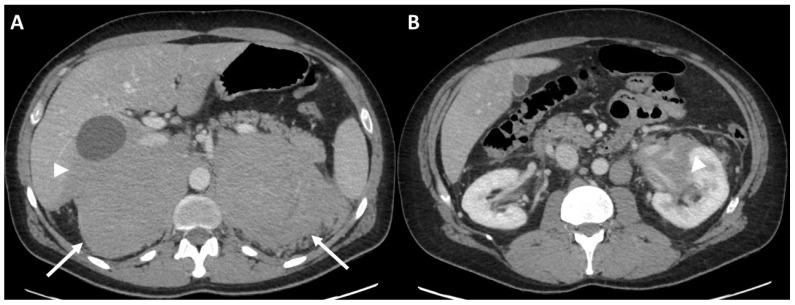
Case #1. Baseline CT images (**A** = bilateral adrenal lesion; **B** = left kidney invasion). The arrows showed the adrenal lesions. The arrowhead showed the invasion in the left renal pelvis.

Clinical data. A 52-year-old man came to our attention after a CT finding of bilateral adrenal lesions (>100 mm) and a hepatic lesion of 90 mm. The examination was performed after the onset of asthenia, abdominal pain and weight loss of 6 Kg within the last 2 months. Blood exams showed hyponatremia, hyperkalemia, normal serum basal cortisol with increased ACTH and renin, while urinary metanephrines were within the range of normality. Hence, we performed a 250 mcg of hexacetate tetracosactide (Synacthen) stimulation test, which demonstrated a reduced adrenal reserve [14]. The patient was therefore hospitalized for further investigation. A biopsy of the hepatic lesion confirmed an infiltration by a high-grade B cell lymphoma with MYC, BCL2 and BCL6 rearrangements. A few days later, the patient underwent the first cycle of CHOP-chemotherapy; however, he was hospitalized again after the occurrence of an adrenal crisis. Unfortunately, the clinical condition worsened and the patient was transferred to the intensive care unit mainly due to hypotension and metabolic acidosis. Despite intensive treatment, he died 10 days later. Baseline ceCT images (Figure 2). Bilateral adrenal masses with irregular margins were identified, with maximum axial diameters of 143 × 102 mm on the right and 139 × 92 mm on the left (Figure 2a; arrows) side. The right adrenal mass was invading the liver while the left one had an extension to the left kidney hilum, also with parenchymal involvement (Figure 2b, arrowhead). The mean densitometry of the masses in the unenhanced scan was 40 Hounsfield Unit (HU) for the right and 39 HU for the left. The enhancement in the arterial and venous phase of the lesions was somehow homogeneous despite the dimensions, without area of necrosis or calcification. The calculated absolute and relative washout of contrast medium were not suggestive for adrenal adenomas. 

**Figure 3:**
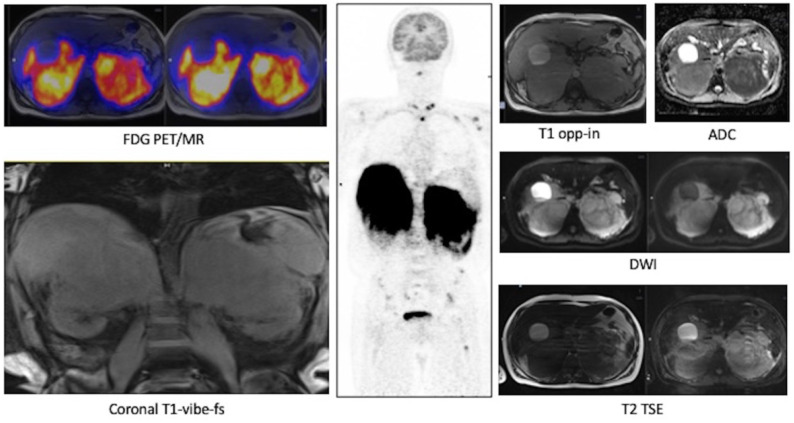
Case #1. FDG PET/MR images.

Baseline FDG PET/MR images (Figure 3). The images showed an intense FDG uptake in both the enlarged adrenal glands (Table 4) and multiple areas of tracer uptake in the bones, compatible with skeletal metastases. In MR images, the signal of the two lesions was mainly homogeneous. Lesions did not show signal drop in out of phase sequences, with signal restriction in diffusion weighted imaging (DWI) sequences, isointensity to liver parenchyma in T1-weighted sequences and slight hyperintensity in T2-weighted images. MR axial and coronal images confirmed the invasion of liver and left kidney parenchyma while on the right side, the adrenal lesion became larger than in the CT scan and an invasion of the right kidney was appreciable. 

#### 3.2.2. Restaging: Case #2 

**Figure 4:**
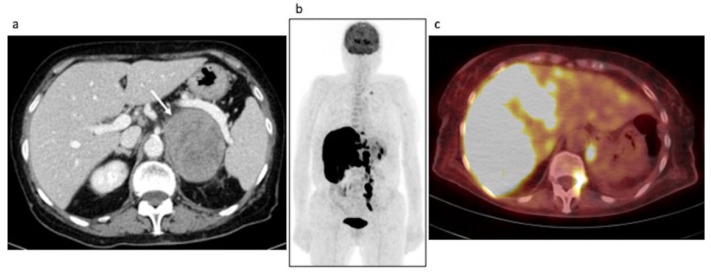
Case #2. Baseline CT (**a**) and post-therapy FDG PET/CT images (**b**,**c**). The arrow illustrated the left adrenal lesion.

Clinical data. An 85-year-old female with a previous history of colon cancer was evaluated after finding a left adrenal lesion during a ceCT performed for oncological follow-up. ceCT images showed a large left adrenal mass with sharp margins and maximum axial diameters of 78 × 63 mm, showing a mean density of 30 HU in the unenhanced scan and with a slight inhomogeneity of the enhancement pattern in the contrast enhanced scans, anyway without areas of necrosis or calcification (Figure 4a, arrow). The contrast medium washout in the late phases was not compatible with the diagnosis of adenoma. The lesion, not visible in a previous CT performed 2 years before, showed an ipsilateral kidney infiltration. The patient reported a 3 Kg weight loss in 2 months while her blood exams revealed hyponatremia, normal basal serum cortisol and increased ACTH; urinary metanephrines were not altered. Despite a 250 mcg Synacthen, a stimulation test was suggested, but it was unperformed. The patient underwent left adrenalectomy, nephrectomy and splenectomy, by laparotomy. Histology was indicative of DLBC-Lymphoma non-GCB (germinal center B-cell) type according to the Hans algorithm. She was discharged with acetate cortisone as replacement therapy. One month later, the patient was hospitalized again because of an adrenal crisis, presented with hyponatremia and dehydration, and intercurrent sepsis. Unfortunately, she died 2 days later. Interestingly, AI occurred despite replacement therapy and hampered the transformation of acetate cortisone into the active form. 

Restaging [18F]FDG PET/CT images (Figure 4b,c). The scan showed an intense pathological FDG uptake in multiple sites of disease (MIP—Figure 4b), such as adrenal lodge, subdiaphragmatic lymph nodes, liver and bone (Figure 4c). 

#### 3.2.3. Evaluation of Response to Therapy: Case #3, Case #4 and Case #5 

##### Case #3 

**Figure 5:**
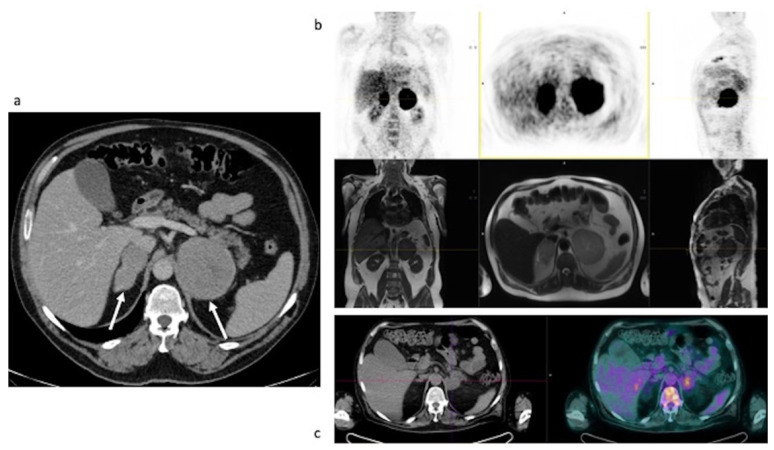
Case #3. Baseline CT (**a**), baseline FDG PET/MR (**b**) and post-therapy FDG PET/CT (**c**). The arrows demonstrated the left and the right adrenal lesions.

Clinical data. A 56-year-old man underwent an abdominal ultrasound for back pain which revealed bilateral adrenal lesions. Abdominal ceCT showed bilateral adrenal masses of 62 × 33 mm on the right and 88 × 80 mm on the left, with a mean density in unenhanced scan of 40 HU on the right side and 39 HU on the left, both showing sharp margins. Even in this case, the enhancement pattern was only slightly inhomogeneous without areas of necrosis or calcification (Figure 5a, arrows). A mediastinal biopsy was indicative of an early T-cell precursor acute lymphoblastic leukemia with FLT3 mutation. Blood exams were suggestive of AI, indeed basal serum cortisol was low, with parallel increase of ACTH. Therefore, hyponatremia and hyperkalemia were detected, and a 250 mcg Synacthen stimulation test confirmed AI. Patient underwent several chemotherapy cycles (LAL protocol) and allogeneic bone marrow transplant. Restaging imaging confirmed a clear regression of adrenal lesions (see PET/MR section). Moreover, adrenal function significantly ameliorated: serum cortisol returned to a normal level, while stimulation test showed an improvement of adrenal reserve despite persistence of subclinical AI. 

Baseline FDG PET/MR and post-therapy FDG PET/CT images. Baseline PET/MR showed a bilateral intense uptake of FDG in both the enlarged adrenal gland (Table 4). No additional site of pathological uptake was found in the other body regions (Figure 5b). Post-therapy PET/CT, performed after 30 days from the last chemotherapy, showed a significant reduction of FDG uptake in both the adrenal gland, with a slight persistent uptake in the left adrenal lesion (Table 4, the uptake in the left adrenal lesion was moderately higher than the liver background, Deauville Score: 4) (Figure 5c). Therefore, a metabolic partial response was reported. However, the residual FDG uptake can be due also to a recovery of a normal adrenal function. Therefore, monitoring of biochemical and clinical parameters is essential in this setting. 

Follow-up. 18F-FDG PET/CT scans were obtained during follow-up (until to 24 months from the end of chemotherapy) to confirm the long-term remission of disease (Supplementary Figure S1).

##### Case #4 

**Figure 6:**
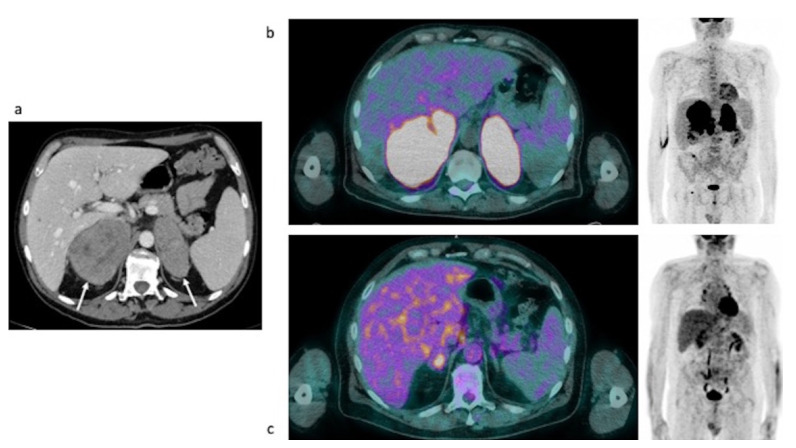
Case #4. Baseline CT (**a**), baseline and post-therapy FDG PET/CT (**b**,**c**).

Clinical data: A 56-year-old man was initially hospitalized in a peripheral hospital because of persistent fever, asthenia and weight loss of 10 Kg in 2 months. A chest-abdominal CT showed bilateral large adrenal lesions with maximum axial diameters of 102 × 73 mm on the right and 90 × 37 mm on the left, with a mean density of 32 HU, sharp margins and an inhomogeneous enhancement pattern especially of the larger nodule (Figure 6a, arrow). The right lesion showed caval vein infiltration with concomitant thrombosis. Blood exams showed normal basal cortisol and aldosterone, with increased ACTH and renin; however, subclinical AI was unmasked performing a 250 mcg Synacthen stimulation test. After excluding increased levels of urinary metanephrines, we performed an adrenal biopsy which was indicative of DLBC-Lymphoma non-GCB type according to the Hans algorithm. Acetate cortisone and fludrocortisone were started as replacement therapy and the patient was treated with lenalidomide R-CHOP (rituximab, cyclophosphamide, vincristine and prednisone). Biochemical parameters sharply improved. Basal and stimulated adrenal function were fully restored, allowing replacement therapy withdrawal. 

Baseline and post-therapy FDG PET/CT images. Baseline PET/CT showed a bilateral intense uptake of FDG in both the enlarged adrenal gland (Table 4). No additional site of pathological uptake was found in the other body regions (Figure 6b). Post-therapy PET/CT, performed after 30 days from the last chemotherapy, showed a significant reduction of FDG uptake in both the adrenal gland, with a persistent moderate uptake in the right adrenal lesion (SUVmax and MTV: 6.03 and 16, respectively, the uptake was moderately higher than the liver background, Deauville Score: 4) (Figure 6c). Therefore, a metabolic partial response was reported. 

Follow-up. CT images were used for monitoring the status of disease, during follow-up. After 2 years from the end of therapy, the remission of disease was reported (Supplementary Figure S2).

##### Case #5 

Clinical data. An 81-year-old man, who lamented confusion and dizziness, was hospitalized in the Endocrinology Unit after finding severe hyponatremia 113 mmol/l (135–145 mmol/l) and high copeptin level at blood exams. Patient was treated with hypertonic saline infusion with poor benefits. Clinical and biochemical features were suggestive for a syndrome of inappropriate antidiuresis (SIAD). The patient was affected by paraneoplastic SIAD considering high blood levels of copeptin and the presence of hematological neoplasm. 

**Figure 7:**
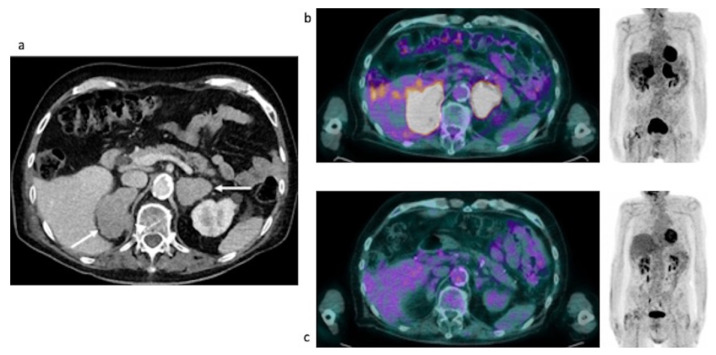
Case #5. Baseline CT (**a**), baseline and post-therapy FDG PET/CT (**b**,**c**). The arrows illustrated the adrenal lesions.

An endocrinological assessment was performed, revealing increased levels of ACTH and renin despite normal serum basal cortisol. However, a 250 mcg Synacthen stimulation test highlighted a subclinical AI. Patients underwent a chest-abdomen ceCT, showing bilateral adrenal lesions with maximum diameters of 70 × 49 mm on the right and 47 × 35 mm on the left, a mean density of 30 HU on the right and 31 HU on the left, irregular margins of the right mass that was invading the diaphragm on the medial side and sharp margins of the left nodule (Figure 7a arrow). The contrast enhancement was substantially homogeneous, and the masses did not show signal drop in the opposed phase sequences (Figure 7a arrow). An adrenal biopsy was performed, revealing a DLBC-Lymphoma GCB type according to the Hans algorithm. The patient was treated with R-COMP (like R-CHOP but doxorubicin replaced by non-pegylated liposomal doxorubicin) that produced a total regression of adrenal lesions and an improvement of adrenal reserve, indicating a clear recovery of hypothalamic–pituitary–adrenal (HPA) axis function. 

Baseline and post-therapy FDG PET/CT images. Baseline PET/CT showed a bilateral intense uptake of FDG in both the enlarged adrenal gland (Table 4). No additional site of pathological uptake was found in the other body regions (Figure 7b). Post-therapy PET/CT, performed after 28 days from the last chemotherapy, showed a complete disappearance of FDG uptake in both the adrenal lesions (Deauville Score = 1) compatible with a complete metabolic response (Figure 7c).

Follow-up. 18F-FDG PET/CT scans were obtained during follow-up (until to 12 months from the end of chemotherapy). However, during the follow-up period, the patient developed a colon-rectal cancer with hepatic metastases. However, serial PET/CT documented the long-term remission of Al (Supplementary Figure S3).

## 4. Discussion

Data about the role of FDG PET/CT or PET/MR in this setting of patients are limited, for two main reasons: the rarity of disease and the absence of specific recommendation. In the present minireview and pictorial essay, we have described five patients affected by PAL who were studied with FDG PET/CT or PET/MR in different phases of disease (staging, restaging and evaluation of response to therapy). Moreover, this is the first paper that assessed the role of PET/MR in PAL. As reported in literature, PET/MR with FDG is the first choice for the imaging of pediatric lymphoma, but due to its high contrast resolution, it would be the first choice also in patients suspicious for PAL. Indeed, it can be useful to detect the presence of the primary adrenal lesions and its widespread in the other organs, mainly in the bone marrow. This is the first paper that reported an experience on PAL with a hybrid PET/MR scanner. 

By the present limited experience, we can assume that the correct diagnostic workup can improve the prognosis of the patients, because of the correct staging of disease and the evaluation of response to therapy. However, further studies, including a high number of patients affected by adrenal lymphoma, are needed to ascertain that. 

In the present experience, we found that FDG uptake was high in all type of Al, although they showed different histological characteristics. This is in line with the current evidence [15]. Moreover, in line with the previously published paper, the adrenal lesions have an elevated glucose metabolism that can be quantified by SUVmax and MTV. In our study, in most cases, the SUVmax was higher than 20 and MTV was more often more than 500. PET/CT quantitative parameters in addition to baseline clinical parameters could be a future direction in PET/CT tailored strategy in patients with lymphoproliferative disease [16]. However, in patients treated with chemotherapy, the metabolic response was either partial or complete. Radiomic analysis would be probably interesting in this setting of disease [17]. In PAL, CT characteristics are not very specific. As reported in some papers, they are often described as hypodense, homogeneous or heterogeneous masses, with a variable density and a moderate enhancement; moreover, in some cases, looking like necrosis or like cystic components [18,19]. In our case series at CT images, the adrenal lesions showed a solid mean densitometry on unenhanced scan, higher than the cut-off of 10 HU commonly used in clinical practice to define the benignity of an adrenal nodule. Moreover, they showed a homogeneous contrast enhancement, despite their dimensions, and that is typical of extra nodal localizations of lymphoma [20,21]. Conversely, MRI features that allow to identify PAL are the low drop of signal intensity in the opposed phase sequences, a heterogeneous hypointensity on T1-weighted sequences and hyperintensity on T2-weighted images with a progressive mild-moderate enhancement after intravenous contrast-medium injection [22,23]. In the early stage of lymphomatous invasion of the adrenal glands, only a uniform enlargement of the glands can be appreciated, which can be confused with an adrenal hyperplasia [23]. Unfortunately, the features described above are not characteristics of PAL since most of the malignant adrenal lesions can present these signs. In our experience, the absence of an extensive area of necrosis inside the lesion and the presence of bilateral adrenal masses can be considered two clues able to tend to the diagnosis of PAL.

Imaging, jointly with endocrine evaluation, is fundamental for the differential diagnosis of adrenal incidentalomas. The most frequent type of adrenal mass is the adenoma (secreting or not), with a reported prevalence up to 80% [5]. Adrenal adenomas show typical features on CT, MRI and PET/CT imaging that allow the radiologists in most cases to identify them correctly. These features are the mean densitometry <10 HU in unenhanced CT scan, high was-out of contrast medium in late phases, signal drop in out-of-phase sequences and low uptake of 18F-FDG tracer [24,25]. Other less frequent neoplastic lesions of the adrenal glands are metastases, lymphoma, pheochromocytoma, myelolipoma, infectious processes and hematomas [5]. Metastases, lymphoma, adenomas and infectious processes are more often bilateral, hence, in the case of bilateral adrenal glands involvement, such type of diseases should be included among differential diagnosis [24].

The present study has some limitations. First, the very small number of patients despite the rarity of the disease. Until now, no more than 200 cases have been reported in the literature [2]. Secondly, the absence of a specific diagnostic workflow could have probably affected the prognosis of cases #1 and #2. Finally, being a retrospective study, no data about long-term prognosis are at present available for cases #3, #4 and #5.

## 5. Conclusions

[18F]FDG-PET should be part of the initial examination of a malignant lymphoproliferative adrenocortical lesion, allowing the staging of the disease. As emerged from the present collection of data, in accordance also with preliminary available data from literature, FDG PET either with CT or MRI can be useful also in the restaging, and especially for the evaluation of treatment response.

## Figures and Tables

**Table 1:** Characteristics of the selected papers.

Authors	Ref	Year of Pub	Country	N of Patients	Scanner Type	Clinical Indication	Analysis of the Images	Main Conclusions
Kasaliwal et al.	[10]	2015	India	5	PET/CT	Baseline and post-therapy	NA	The conclusions were not relative to FDG PET/CT.
Laurent et al.	[12]	2017	France	10	PET/CT	Initial staging	Visual and SUVmax	PET can visualize extra-adrenal locations of disease, more than CT.
Altinmakas et al.	[11]	2019	USA	6	PET/CT	Initial staging	Visual and SUVmax	Adrenal lymphoma has a high FDG uptake.
Wang et al.	[13]	2019	China	8	PET/CT	Initial staging	PET metrics (also radiomics)	Texture analysis may be used for the prediction of OS.
Majidi et al.	[9]	2020	USA	18	PET/CT	Initial staging	Visual and SUVmax	PET can visualize extra-adrenal locations of disease, more than CT.

NA: not available.

**Table 2:** QUADAS 2 in all selected studies.

Authors, Ref	Risk of Bias				Applicability Problems		
	Patient Selection	Study Test	Standard of Reference	Flux and Timing	Patient Selection	Study Test	Standard of Reference
Kasaliwal et al. [10]	Unclear	Low	Low	Unclear	Unclear	Low	Low
Laurent et al. [12]	High	Low	Unclear	Unclear	High	Low	Unclear
Altinmakas et al. [11]	Unclear	Low	Unclear	Unclear	Unclear	Low	Unclear
Wang et al. [13]	Low	Low	Low	Low	Low	Low	Low
Majidi et al. [9]	Low	Low	Low	Low	Low	Low	Low

**Table 3:** Endocrinological parameters collected before chemotherapy (CT) and after at least 6 months of CT.

Endocrine Parameters	Patient #1	Patient #2	Patient #3		Patient #4		Patient #5	
	Before Imaging	Before Imaging	Before Imaging	After Chemotherapy	Before Imaging	After Chemotherapy	Before Imaging	After Chemotherapy
ACTH (10–50 ng/l)	319	145	105	97	129	30	89.4	56.1
Serum cortisol (138–690 nmol/l)	291	378	90	326	262	354	277	396
Na (136–145 mmol/l)	112	121	132	143	136	140	113	137
K (3.4–4.5 mmol/l)	6.8	4.8	4.9	3.7	4.1	4.5	5.4	4.4
Cortisol post Synacthen test t0′→ t60′ (>500 nmol/l)	363→374	NA	306→339	285→461	252→337	480→563	326→333	378→470
Renin (4.4–46.1 mIU/l)	85.1	2.2	30.3	NA	72.4	38	262.4	161.8
Aldosterone (70–1086 pmol/l)	168	61.9	138	NA	156	297	62.2	281

NA: not available.

**Table 4:** PET metric in all selected patients.

		Patient #1	Patient #2	Patient #3	Patient #4	Patient #5
Baseline	SUVmax (LAG)	20.3	NA	19.7	30	48.43
	SUVmean (LAG)	6.73	NA	5.09	4.37	9.8
	MTV (LAG)	2393.94	NA	800	960.35	75.99
	SUVmax (RAG)	21.3	NA	21.6	28.3	53.22
	SUVmean (RAG)	6.98	NA	4.82	4.88	4.88
	MTV (RAG)	3310.61	NA	344.94	1348.02	255.43
Post-therapy	SUVmax (LAG)	NA	NA	4.96	no uptake	no uptake
	SUVmean (LAG)	NA	NA	2.48	no uptake	no uptake
	MTV (LAG)	NA	NA	26.02	no uptake	no uptake
	SUVmax (RAG)	NA	NA	no uptake	6.03	no uptake
	SUVmean (RAG)	NA	NA	no uptake	2.43	no uptake
	MTV (RAG)	NA	NA	no uptake	16.17	no uptake
Metabolic response		-	-	PMR *	PMR *	CMR *

NA: not available; * Lugano Criteria (Deauville Score); PMR = partial metabolic response; CMR = complete metabolic response.

## Supplementary Materials

The following supporting information can be downloaded at: https://www.mdpi.com/article/10.3390/curroncol29100623/s1, Figure S1: Serial FDG PET/CT scans during follow-up (FUP) in case #3; Figure S2: Serial contrast-enhanced computed tomography (ceCT) scans in case #4; Figure S3: Serial FDG PET/CT scans (from baseline to the last imaging follow up-FUP) in case #5; Table S1: Histopathogical and Immunohistochemical features of the adrenal lymphoma for each case.
