# Supplementary material for: [18F]FDG PET/CT and PET/MR in Patients with Adrenal Lymphoma: A Systematic Review of Literature and a Collection of Cases
Source: Curr Oncol. 2022 Oct 18;29(10):7887–99. doi: 10.3390/curroncol29100623 (PMC9600011; doi:10.3390/curroncol29100623)
Supplement: Supplementary file 1 [file curroncol-29-00623-s001.zip › curroncol-1954026-supplementary.pdf]

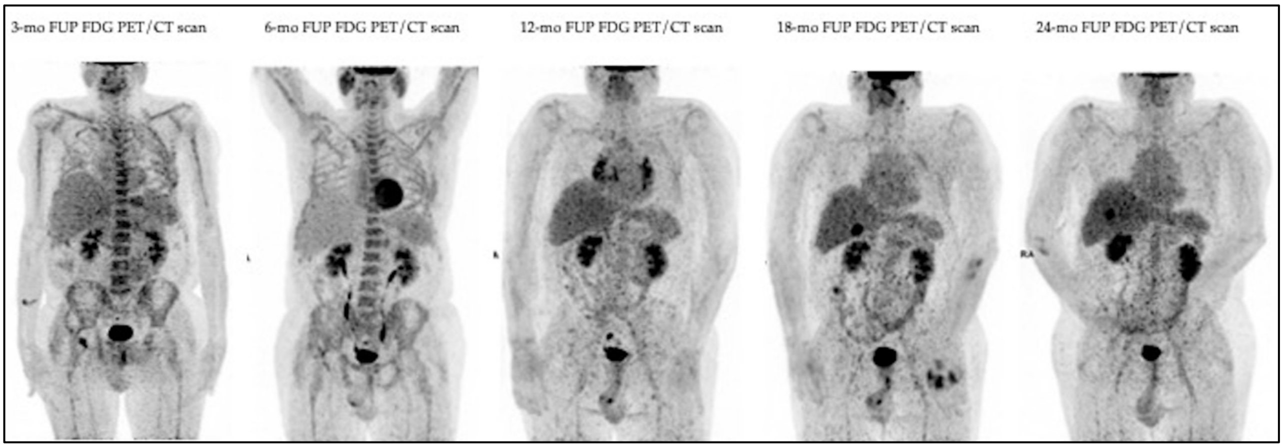

Figure S1. Serial FDG PET/CT scans during follow-up (FUP) in case #3.

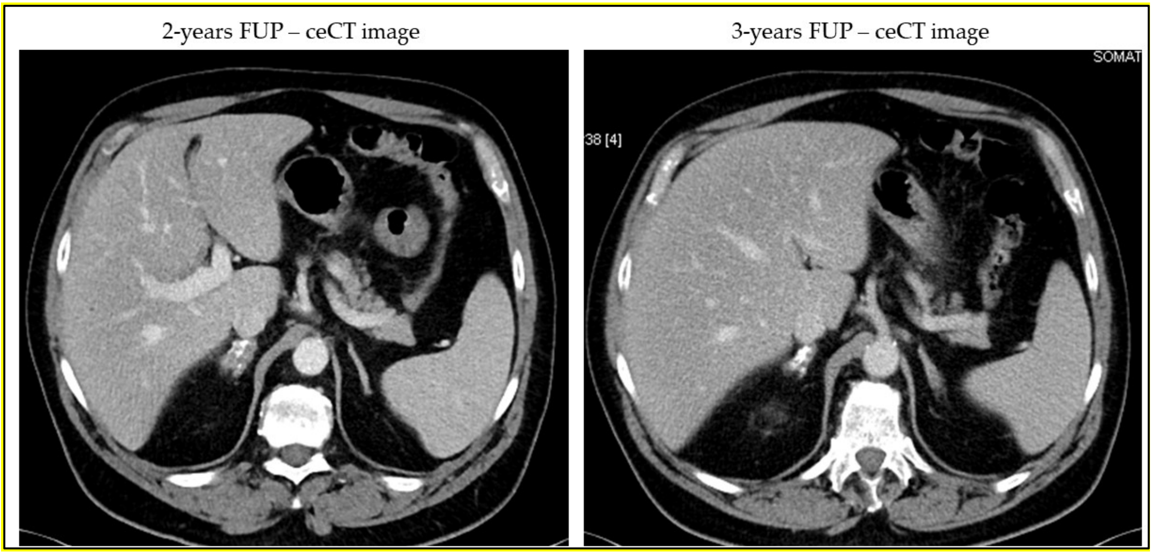

Figure S2. Serial contrast-enhanced computed tomography (ceCT) scans in case #4.

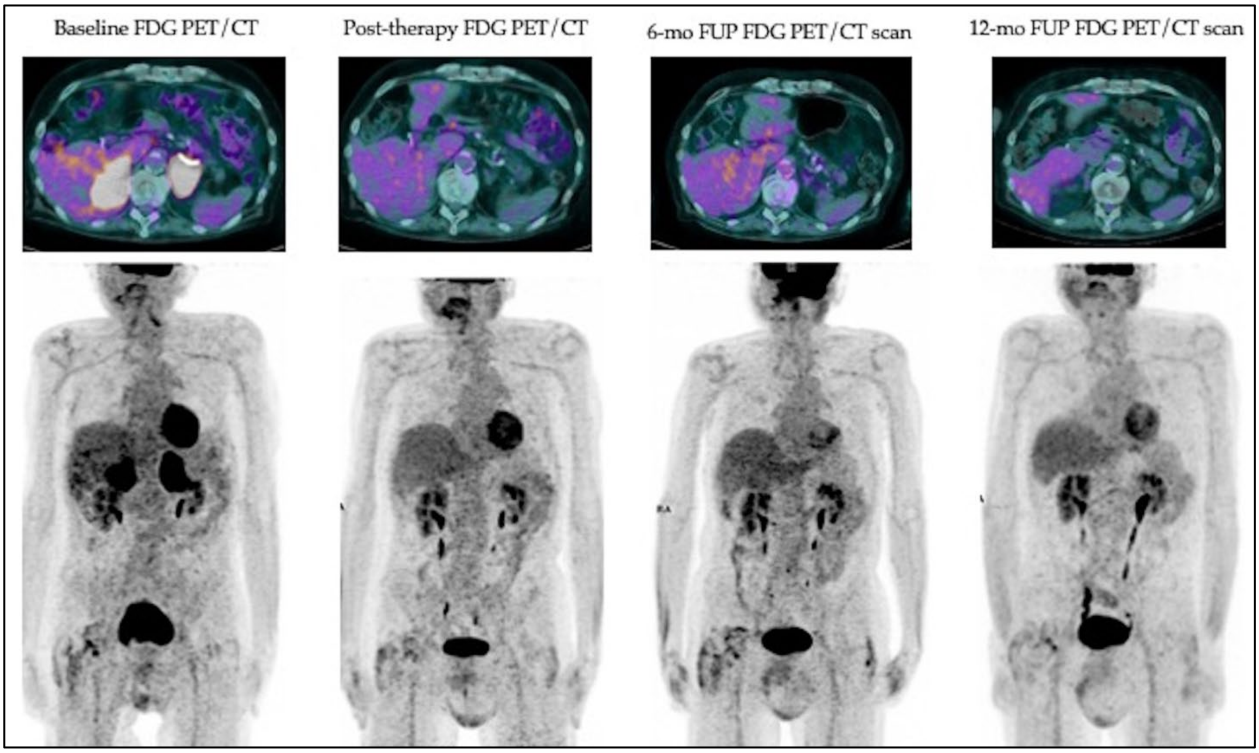

Figure S3. Serial FDG PET/CT scans (from baseline to the last imaging follow up-FUP) in case #5.

Table S1. Histopathogical and Immunohistochemical features of the adrenal lymphoma for each case.

Case	Site of Biopsy	Histopathological Characteristics	Immunohistochemical Characteristics	Final Diagnosis Based on Hans Algorithm
#1	Needle biopsy of the liver nodules	Lymphoid cells with diffuse growth patterns, characterized by medium-size mitotically active cells with necrosis	CD20+, CD-, CD10+, BCL6+, MUM1+, c-Myc+, BCL2+, Cyclin D1-, TdT-, CD138-, Ki67 95-100%.<br>Cytogenetic by FISH reveled rearrangement of MYC (68% of 100 analyzed cells), BCL2 (0% of cells) and BCL6 (92% of cells)	High-grade B cell lymphoma
#2	Left adrenalectomy and nephrectomy	Nort available	CD20+, CD3-, CD10-, MUM1+, Cyclin D1-, BCL2+, BCL6+, c-Myc+, TdT-, EBER-, Ki67 90%, D45+, MDM2-, S100, AE1/AE3-	Diffuse large B-cell lymphoma non-GCB type
#3	Biopsy of the mediastinal mass	With connective sclerotic tissue there are small to medium size lymphocyte and fine chromatin, irregular nuclear border, and high nucleus/cytosol ratio	CD3+, CD2-, CD5-, CD7+, CD4+/-, CD8-, TdT-, CD1a-, CD34-, CD99+, CD33-, CD30-, CD20-, CD56-, CD57-, EBER-, Ki67 70-80%, CD117+, CD10-, MPO-.<br>Molecular analysis found a mutation in FLT3 gene (FLT3-ITD)	T-cell precursor lymphoblastic and leukemia
#4	Needle biopsy of the left adrenal gland	Not available	CD20+, CD3-, CD5-, CD10-, BCL2+, BCL6+, CD30-, ALK-, MUM1+, Ki67 70-80%	diffuse large B-cell lymphoma non-GCB type
#5	Needle biopsy of the left adrenal gland.	Diffuse infiltration of large-size mitotically active lymphocyte with eosinophils and plasma cells	CD20+, CD3-, CD10+, BCL6+, MUM1+, BCL2-, c-Myc-, CD30-, Cyclin D1-, TdT-, EBER-, Ki67 75%<br>Cytogenetic by FISH did not find rearrangements of MYC	Diffuse large B-cell lymphoma GCB type
